# The indirect impact of COVID-19 pandemic on limb preservation care– a retrospective analysis of trends in lower limb revascularisation

**DOI:** 10.1186/s13047-023-00648-6

**Published:** 2023-08-09

**Authors:** Lakmali Anthony, Madeline Gillies, Morica Tran, David Goh

**Affiliations:** 1 Department of Vascular Surgery, Northern Health, 185 Cooper Street, Epping, VIC 3076 Australia; 2 Department of General Surgery, Goulburn Valley Health, 2-48 Graham Street, Shepparton, VIC 3630 Australia; 3 Department of Podiatry, Austin Health, 145 Studley Road, Heidelberg, VIC 3084 Australia

## Abstract

**Background:**

Disruptions caused by COVID-19 pandemic have profoundly influenced the management of many conditions, especially vascular pathologies including limb preservation care. The aim of this study is to evaluate the impact of the pandemic on patients with peripheral arterial disease (PAD) focusing on lower limb revascularisation procedure volume, their indication and urgency of surgery.

**Methods:**

The Australian Vascular Audit (AVA) was used to capture data on revascularisation procedures before and after the onset of the pandemic in Victoria, Australia. Information on patient demographics, procedures performed, their indication and urgency of surgery were collected.

**Results:**

There was a significant 22.7% increase in revascularisations for PAD during the COVID-19 pandemic, driven solely by a 31.9% increase in endovascular revascularisation procedures. Revascularisation procedures for all indications of PAD, namely claudication, rest pain and tissue loss, increased by 14.8%, 39.2% and 27.4% respectively, during the pandemic compared to pre-pandemic times. Open procedures declined by 10.2% during the pandemic. There were significant 13.9% and 62.2% increases in elective and semi-urgent revascularisations respectively during the pandemic while emergency revascularisations for PAD fell by 4.2%. There were no significant increases in toe, forefoot or below knee amputations during the pandemic compared to pre-pandemic times.

**Conclusions:**

This study found that the volume of revascularisation for PAD increased significantly during the pandemic indicating that patients with PAD had significant deterioration of their condition during the pandemic. This is likely multifactorial; due to disruptions to standard provision of podiatry, vascular surgery and endocrinology services to these patients, a decline in overall health and changes in health-related behaviours due to restrictions and infection control methods imposed during the pandemic. The number of elective and semi-urgent procedures also increased during the pandemic which reflects the significant deterioration of PAD patients during the pandemic. This study highlights a concerning trend of worsening PAD when routine care of these patients is disrupted. Such data should be instrumental in contingency planning and resource allocation for managing the ongoing pandemic.

## Introduction

Peripheral arterial disease (PAD) is a growing problem due to the aging population and the current global epidemic of diabetes [1]. PAD is a condition that results from obstruction of blood flow in the arteries of the lower limbs. Intermittent claudication, defined as pain in the calves or thighs during ambulation, is one of the earliest symptoms. As PAD progresses in severity, patients experience pain at rest, particularly at night when the legs are elevated in bed, and relieved by dependency. In the advanced stages of PAD, tissue loss in the form of ischaemic ulceration and gangrene occurs due to persistent and prolonged tissue hypoperfusion. The late stages of PAD, namely when the disease progresses to rest pain and tissue loss, are collectively referred to as chronic limb-threatening ischaemia (CLTI) [2]. Amputation is usually required if CLTI is not diagnosed and treated promptly. Importantly, CLTI carries a one-year mortality rate of approximately 20% [3, 4].

Patients with PAD require a multidisciplinary approach to management involving podiatrists, vascular surgeons and endocrinologists to deliver appropriate limb preservation care. They often need ongoing revascularisation procedures to maintain limb perfusion; therefore, it is vital to continually monitor their tissue loss and vascular status, a service usually provided by podiatrists. Vascular surgeons and endocrinologists manage revascularisation and diabetes care, respectively. Revascularisation can be done with open surgery or with endovascular procedures using balloons and stents. Diabetes is a significant risk factor for PAD, and adequate control of blood sugars is vital in preventing disease progression. PAD patients are often seen in multidisciplinary clinics, with all three specialties providing limb preservation care.

The disruptions to health service delivery that have accompanied the COVID-19 pandemic has undoubtedly had a profoundly negative impact on patients with PAD due to delays in routine follow-up of patients and cancellation of elective surgery. Additionally, barriers caused by requirements of self-isolation and lockdowns have also negatively impacted many patients with chronic diseases such as PAD.

Many studies from around the world report an increase in lower limb amputations during the pandemic compared to pre-pandemic times [5]. However, there is limited evidence on the impact of the pandemic on PAD patients, particularly those requiring revascularisation procedures.

An early study from Australia and New Zealand reported a significant decrease in vascular surgery procedures in 2020 compared to 2015–2019 [6]. Another study from Switzerland reported a reduction in the number of vascular surgery procedures in 2020 compared to 2018, but the proportion of patients requiring surgery for acute limb ischaemia was higher during the pandemic [7]. Another study from Romania showed a significant decrease in non-urgent vascular surgery during the pandemic [8]. Other studies have also reported that patients with PAD who presented to hospital during the pandemic, often presented with severe disease compared to pre-pandemic times and required more extensive revascularisations [9–12]. These studies have mainly focused on the first wave of the pandemic. However, since 2020, the pandemic has progressed with ongoing disruptions to the standard provision of health care. The state of Victoria in Australia had some of the harshest COVID-19-related restrictions in the world, with the last lockdown being lifted in December 2021. By December 2021, Victorians have spent a cumulative 263 days in lockdown [13].

As such, further studies are required to investigate the long-term effects of the ongoing pandemic on patients with PAD. This study aims to evaluate the impact of the pandemic on patients with PAD by analysing lower limb revascularisation procedure volume for PAD, their indication and the urgency of surgery during the pandemic compared to pre-pandemic times.

## Methods

### Data collection

This retrospective cohort study used deidentified patient data from the Australasian Vascular Audit (AVA). To maintain specialist registration with the Australian and New Zealand Society for Vascular Surgery (ANZSVS), vascular surgeons practising in both countries are required to capture a minimum dataset relating to all lower limb amputations as well as open bypass operations and endovascular interventions performed for lower limb PAD [14]. AVA data include only patients who undergo a procedure and are collected at two points during each hospital admission episode; at the procedure and at discharge. AVA data are collected via a web-based application with each data field coded to a limited categorical response variable to ensure consistency. In addition, the data are subject to both internal and external validation, which has been published previously [14]. All adult patients who underwent an open or endovascular lower limb revascularisation procedure for claudication, rest pain or tissue loss in Victoria, Australia between July 1 2018 and December 31 2021 were included. The date of imposition of strict lockdown in Victoria, Australia (March 31 2020) was used to divide the cohort into a pre- and post-lockdown groups. Procedures performed for acute limb ischaemia, arterial dissection, vascular entrapment, aneurysmal and neoplastic disease were excluded. Data collected included demographic data from deidentified patients (age, sex) and comorbidities, including the presence of diabetes mellitus, ischaemic heart disease (IHD), chronic kidney disease (CKD), and hypertension. In addition, procedural data were captured including the date of procedure, operative approach (open bypass and endarterectomy or endovascular intervention), the indication for revascularisation, and the urgency (coded as elective, semi-urgent or emergent). The Royal Australasian College of Surgeons (RACS) Ethics Committee provided ethical approval for AVA data collection and use in research publications. As such, individual patient and local hospital ethics committee ethical approval was not required.

### Statistical analysis

Descriptive statistics were used to summarise demographic, comorbidity, and operative data. Age (years) was recorded as a continuous variable and presented as mean and standard deviation; all other variables were categorical and reported as frequency and valid percentage. Student’s t test and one-way ANOVA were used to analyse the relationship between dependent variables and age, and a chi-square test was used to assess the relationships between categorical variables. An α level was determined a priori and *p*-values less than 0.05 were considered statistically significant. Statistical analysis was performed using IBM SPSS Statistics version 29.0 (IBM Corp: Armonk, NY, USA).

## Results

### Patient characteristics

Patient demographic and comorbidity data are presented in Table 1. The mean age was 72.4 years and more than two thirds of patients were male (*n* = 4414, 71.69%). Age was significantly associated with open approach and semi-urgent and emergency revascularisation (*p* =  < 0.001) (Table 1). Male sex was also significantly associated with open approach (χ^2^ = 34.11,* p* < 0.001) but was unrelated to urgency of revascularisation (Table 1). In terms of comorbidities, 52.59% of patients had ischaemic heart disease, 59.10% had diabetes mellitus, 12.32% had hypertension and 94.6% had chronic kidney disease (Table 1). There was a statistically significant association between the presence of each recorded comorbidity and urgency of operation, while only diabetes and chronic kidney disease were associated with operative approach (Table 1).

**Table 1:** Cohort demographic and comorbidity characteristics and simple univariate analysis association with revascularisation approach, indication and urgency

		**Open**		**Semi-urgent & Emergency**	
	**N**	**χ**^**2**^	***p-*value**	**χ**^**2**^	***p-*value**
Age, mean (SD)^a^	72.4 (11.5)	*F* = 72.02	< 0.001	*F* = 7.57	< 0.001
Male sex	4414 (71.6%)	34.11	< 0.001	7.69	0.053
*Comorbidities*					
IHD	3239 (52.59%)	1.27	0.260	113.58	< 0.001
Diabetes	3634 (59.10%)	27.83	< 0.001	522.11	< 0.001
Hypertension	759 (12.32%)	0.30	0.587	31.95	< 0.001
CKD	5825 (94.6%)	22.37	< 0.001	255.53	< 0.001
*Total*	6518				

*SD* Standard deviation

^a^One-way ANOVA

### Impact of COVID-19 on patient characteristics

Age remained similar in both the pre- and post-lockdown periods (Table 2). There was an increased proportion of female patients in the post-lockdown group (χ^2^ = 4.10, *p* = 0.044) (Table 2). The prevalence of diabetes, chronic kidney disease, and hypertension remained similar between the two groups (Table 2), while the prevalence of ischaemic heart disease increased significantly (χ2 = 6.78, *p* = 0.009) (Table 2).

**Table 2:** Comparison of patient characteristics pre-and post-lockdown

	**Pre-lockdown, n (%)**	**Post-lockdown, n (%)**	**χ**^**2**^	***p-*value**
Age, mean(SD)^a^	72.4 (11.3)	72.4 (11.8)	*t* = 0.054	0.948
*Sex*				
Female	907 (31%)	1197 (33.3%)	4.10	0.044
Male	2020 (69%)	2394 (66.6%)		
*Comorbidities*				
Diabetes	1425 (48.6%)	1814 (50.5%)	2.10	0.147
IHD	1683 (57.5%)	1951 (54.3%)	6.78	0.009
CKD	357 (12.2%)	402 (11.2%)	4.13	0.127
Hypertension	2622 (89.6%)	3203 (89.2%)	0.320	0.527

*SD* Standard deviation, *IHD* Ischaemic heart disease, *CKD* Chronic kidney disease

^a^Student’s t test

### Impact of COVID-19 on procedural characteristics

There were 6,518 revascularisation procedures performed by vascular surgeons in Victoria, Australia between July 1 2018 and December 31 2021 (Table 3). Of these, 3,591 were performed in twenty-one months after the lockdown restrictions were imposed, which was a 22.7% increase from the 2,927 revascularisation procedures performed in the preceding period (χ^2^ = 5883.75, *p* < 0.001). This overall increase was composed entirely of endovascular procedures (34.09% increase), as the number of open procedures decreased by 10.2% in the same period (χ^2^ = 53.08, *p* < 0.001) (Table 3). In terms of indication for surgery, there was a statistically significant increase in the number of revascularisation procedures performed for claudication (χ2 = 5.45, *p* = 0.020) and rest pain (χ2 = 4.19, *p* = 0.041). The number of procedures performed for tissue loss was not significantly different between the time periods (Table 3). Elective and semi-urgent procedures both increased significantly in the post-lockdown period (χ2 = 23.15, *p* =  < 0.001, and χ2 = 37.17, *p* < 0.001, respectively), while emergency procedures did not differ significantly between time periods (Table 3).

**Table 3:** Comparison of pre- and post-lockdown lower limb revascularisation procedures by approach and indication

	**Pre-lockdown, n**	**Post-lockdown, n (%change)**	**χ2**	***p*-value**
Total revascularisations	2927	3591 (+ 22.7%)	5883.75	< 0.001
*Approach*				
Open	677	574 (-15.3%)	53.08	< 0.001
Endovascular	2250	3017 (+ 34.1%)		
*Indication*				
Claudication	1311	1505 (+ 14.8%)	5.449	0.020
Rest pain	388	540 (+ 39.2%)	4.192	0.041
Tissue loss	1197	1524 (+ 27.4%)	1.581	0.209
*Urgency*				
Elective	2199	2505 (+ 13.9%)	23.15	< 0.001
Semi-urgent	585	949 (+ 62.2%)	37.17	< 0.001
Emergency	143	137 (-4.2%)	4.50	0.034

### Trends in lower limb revascularisation procedure volume

**Fig. 1:**
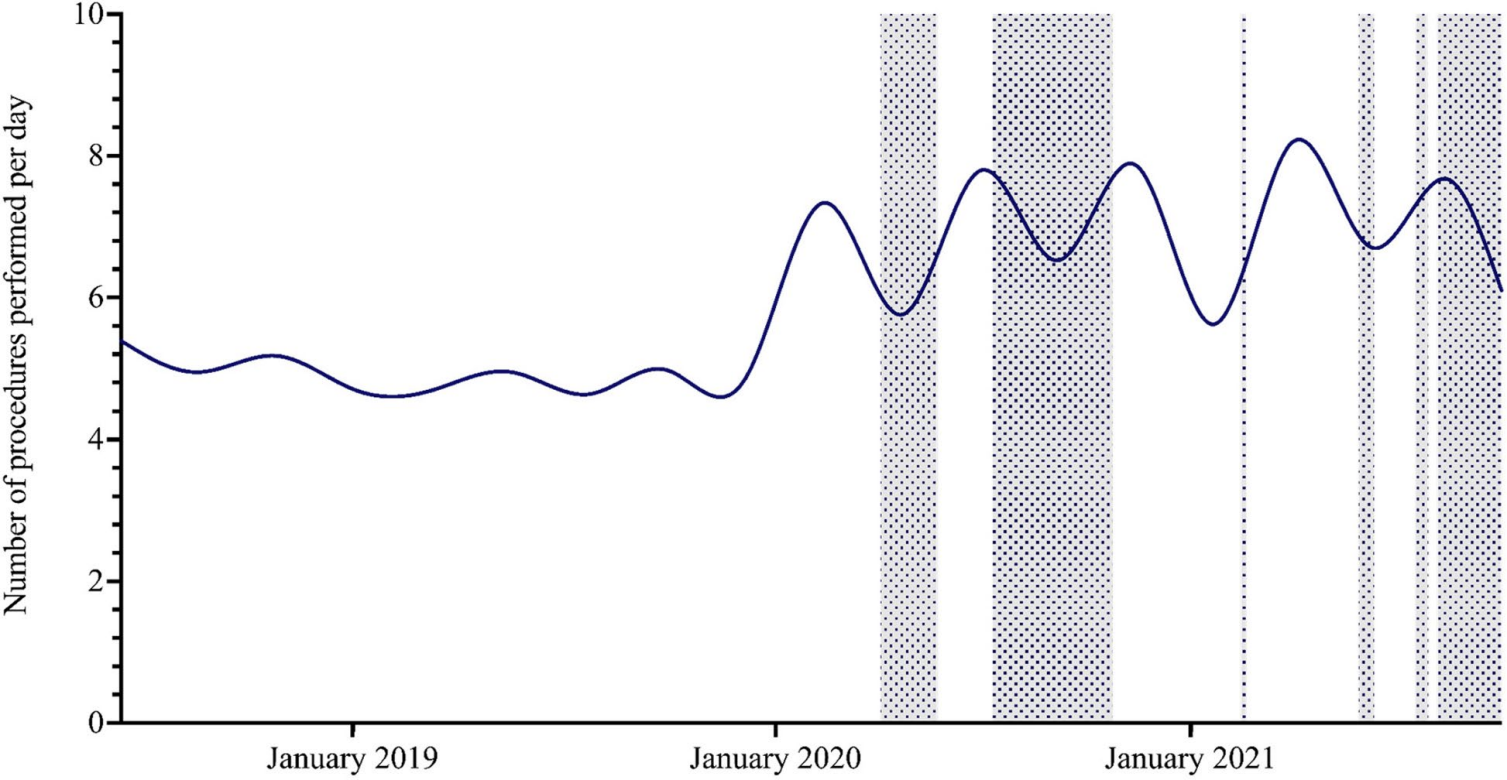
Trends in revascularisation procedure volume (expressed as procedures per day) over the study period. Shaded areas indicate lockdown periods in Victoria

Throughout the study period, the state of Victoria experienced a total of six lockdowns, each with varying durations: Lockdown 1, from March 31st 2020 to May 12th 2020 (43 days); Lockdown 2, from July 9th 2020 to October 27th 2020 (111 days); Lockdown 3, from February 13th 2021 to February 17th 2021 (5 days); Lockdown 4, from May 28th 2021 to June 10th 2021 (14 days); Lockdown 5, from July 16th 2021 to July 27th 2021 (12 days); and Lockdown 6, from August 5th 2021 to October 21st 2021 (77 days) (13). The study found a marked decrease in revascularisation procedures during lockdown periods lasting over 30 days during the pandemic (Fig. 1).

### Lower limb amputations

During the pandemic, below knee amputations (BKAs) increased from 272 to 318, a 16.9% increase. In minor amputations, forefoot amputations decreased from 236 to 228, a 3.4% decrease and toe amputations were roughly similar in the two groups – 1140 in the pre-pandemic group compared to 1152 in the pandemic group, a 1% increase. None of the observed changes in these subgroups of amputations were statistically significant (Table 4).

**Table 4:** Number of amputations in pre-pandemic and pandemic groups

	**Pre-pandemic**	**Pandemic**	**% change**	***p* value**
BKA^a^	272 (15%)	318 (17%)	16.9%	0.058
Minor^a^	1376 (78%)	1379 (75%)	0.22%	0.954
Forefoot^a^	236 (13%)	228 (12%)	-3.4%	0.710
Toe^a^	1140 (64%)	1152 (62%)	1.1%	0.802

^a^Chi square

## Discussion

Lower limb revascularisation is vital for reducing the morbidity associated with PAD and preventing amputations. Some studies have evaluated the impact of the COVID-19 pandemic on PAD focusing on the first wave of the pandemic. The aim of this study was to evaluate the impact of the pandemic on patients with PAD by focusing on lower limb revascularisation procedure volume, their indication and urgency of surgery during a nearly two-year course of the pandemic.

The key findings of this study can be summarised as follows: (i) there was an overall increase in revascularisation procedures for PAD during the pandemic solely driven by an increase in endovascular revascularisation procedures. (ii) there was a decrease in open revascularisation overall and a significant reduction in open procedures done for claudication (iii) there were significant increases in endovascular revascularisation for all stages of PAD, namely claudication, rest pain and tissue loss (iv) there were significant increases in elective and semi-urgent procedures with a reduction in emergency revascularisation procedures (v) there were no significant differences in toe, forefoot or below knee amputations between the pre-pandemic and pandemic groups.

One key finding of this study is the significant increase in endovascular revascularisation procedures. Endovascular revascularisations are minimally invasive procedures that can be done as day cases without the need for many resources from the hospital such as an overnight bed or prolonged nursing care. Therefore, at a time when resources are scarce, it appears that the individual units have attempted to maximise their service delivery with minimal impact on hospital resources. This study however found an increase in endovascular procedures to a far greater degree than what is required to compensate for the decline that was observed in open procedures. There were significant increases in the rate of revascularisation for all indications of PAD, with revascularisations for rest pain and tissue loss increasing by 62.9% and 35% respectively. This is a far greater increase than what is required to compensate for the 10% reduction observed in open procedures. This finding is significant because it indicates that a lot more patients with PAD had deteriorations in their condition during the pandemic requiring revascularisation compared to the pre-pandemic times. This deterioration is likely multifactorial. Firstly, patients with PAD require continuous medical care and require regular follow up with podiatrists, vascular surgeons and endocrinologists for optimal management of their PAD. During the pandemic, many regular follow-up of patients were postponed. Indeed, reports from Australia and around the world indicate clinics could not accommodate as many patients during the pandemic but managed to preserve quality [15, 16]. Secondly, there were changes in patient health-related behaviour with a hesitancy to attend hospitals due to fear of contracting the virus. This reduction in follow-up most likely contributed in a significant way to the deterioration of PAD in these patients which eventually lead to them requiring more revascularisation procedures during the pandemic. Indeed a few studies report that patients who presented to hospital with PAD during the pandemic had more severe disease on average compared to pre-pandemic times and required more advanced interventions or even amputations [9–12]. Another contributing factor to the increase in revascularisations during the pandemic is a general decline in patient health during the pandemic. This has been reported by several studies and a systematic review which found reduced physical activity and increased sedentary behaviours in several populations of adults with a variety of medical conditions during lockdowns [17–19]. Additionally, reduced physical activity and increased sedentary behaviours are associated with poor glycaemic control which overtime likely contributed to deterioration of PAD further in diabetic patients [20, 21].

This finding of increased revascularisations however, is not consistent with the existing, albeit scarce, literature on the topic where a decline in revascularisation for PAD has been observed during the pandemic [22–24]. However, many of these studies only evaluated the first wave of the pandemic therefore a potential increase in numbers due to delayed presentations and referrals cannot be excluded. One study has evaluated the long-term impact of the pandemic on PAD and found similar results of increased revascularisations during the pandemic [9].

Despite an increase in revascularisation procedures likely indicating a deterioration of PAD during the pandemic, this study did not find any statistically significant increases in toe, forefoot or below knee amputations during the pandemic compared to pre-pandemic times. This finding contrasts with what has been reported in other countries [5, 25–31]. In Italy, a threefold increase in amputations has been reported [25]. Similarly, a > 50% increase in major amputations has been reported in India [28]. Given that Victoria had some of the harshest restrictions during the pandemic, it is worth investigating strategies that podiatry and vascular surgery units in Victoria used to somewhat reduce the effects of the pandemic on their services. No studies exist that explore such strategies yet.

This study also found a decline in emergency revascularisations and a significant increase in both elective and semi-urgent procedures for PAD. Notably, semi-urgent procedures increased by 62% during the pandemic. This is expected because emergency revascularisation is usually reserved for acute limb ischaemia. The increase in both elective and semi-urgent procedures fits with a trend of a PAD population with worsening of their condition during the pandemic. The increase in elective procedures likely reflects individual units appropriately prioritising these patients during times when elective surgeries were permitted.

In terms of trends in procedure volume with respect to lockdown periods, this study found a decline in revascularisation procedures during lockdown periods lasting over 30 days, however, over the longer term, there was an overall increase in revascularisation procedures performed during the pandemic. Previous studies have noted a decline in vascular surgery procedures during the pandemic compared to pre-pandemic times, but none have investigated the correlation between procedure volume and lockdown periods or the nature of lockdowns [9–12]. Future studies analysing this correlation could aid in developing lockdown strategies that minimise disruptions to healthcare services.

## Conclusion

This study found that the volume of revascularisation for PAD increased significantly during the pandemic indicating that patients with PAD had significant deterioration of their condition during the pandemic. This is likely multifactorial; due to disruptions to standard provision of podiatry, vascular surgery and endocrinology services to these patients, a decline in overall health and changes in health-related behaviours due to restrictions and infection control methods imposed during the pandemic. The number of elective and semi-urgent procedures also increased during the pandemic which reflects the significant deterioration of PAD patients during the pandemic. This study highlights a concerning trend of worsening PAD when routine care of these patients is disrupted. Such data should be instrumental in contingency planning and resource allocation for managing the ongoing pandemic.

## Data Availability

The data that support the findings of this study are available from Australasian Vascular Audit but restrictions apply to the availability of these data, which were used under license for the current study, and so are not publicly available. Data are however available from the authors upon reasonable request and with permission of Australasian Vascular Audit.

## Abbreviations

PAD: Peripheral arterial disease
COVID-19: Coronavirus disease caused by the SARS-CoV-2 virus
AVA: Australasian Vascular Audit
ANZSVS: Australian and New Zealand Society for Vascular Surgery
